# An In Silico Study Investigating Camptothecin-Analog Interaction with Human Protein Tyrosine Phosphatase, SHP2 (PTPN11)

**DOI:** 10.3390/ph16070926

**Published:** 2023-06-26

**Authors:** Donald Bajia, Katarzyna Derwich

**Affiliations:** Department of Pediatric Oncology, Hematology and Transplantology, Poznan University of Medical Sciences, Ul. Fredry 10, 61701 Poznan, Poland

**Keywords:** camptothecin, novel analogs, SHP2/PTPN11, Verify 3D, ERRAT, molecular docking, in silico approach, protein-ligand interactions

## Abstract

The human PTPN11 gene encodes for the src tyrosine phosphatase protein (SHP2) is now gaining much attention in many disorders, particularly its oncogenic involvement in many types of cancer. Efforts in developing molecules targeting SHP2 with high efficacy are the future of drug discovery and chemotherapy. However, the interaction of a new camptothecin analog with the catalytic domain of SHP2 protein remains unknown. Therefore, this study aims to provide in silico rationale for the recognition and binding of FL118 and irinotecan with the catalytic domain of human protein tyrosine phosphatase-SHP2 (PTPc-SH2-SHP2, chain A). The docking interaction of the human SHP2 protein’s catalytic domain as well as Y279C and R465G mutants with FL118 and irinotecan ligands were calculated and analyzed using the Autodock 4.2 programme, setting the docking grid to target the protein’s active site. The camptothecin analog FL118 had the best lowest negative affinity energies with PTPc-SHP2 wildtype and SHP2-Y279C mutant model (−7.54 Kcal/mol and −6.94 Kcal/mol, respectively). Moreover, the protein-ligand complexes revealed several hydrogen bond interactions reflecting the degree of stability that each structure possesses, with the FL118-SHP2-wildtype forming the most stable complex among the structures. In addition, the FL118-SHP2 wildtype complex was validated for RMSD, RMSF, hydrogen bonds, and salt bridges. This revealed that the complex generated became stable over time. This in silico rationale identifies the novel FL118 camptothecin analog as a potent selective inhibitor of PTPc-SH2 domain of SHP2 protein, paving way for further in vitro investigations into the interactions and binding activity of analogs with SHP2 for potential therapeutic applications in PTPN11-associated disorders.

## 1. Introduction

Protein tyrosine phosphorylation or dephosphorylation are essential physiological signalling processes essential for cell growth, proliferation, differentiation, and migration [1]. Protein kinases (PTK) and phosphatases such as PTP are responsible for reversing phosphorylation, and a homeostatic balance is required under physiological conditions to keep an organism’s system functioning. However, alterations in tyrosine phosphorylation signalling have led to the development of several pathological conditions including neurological abnormalities, cardiomyopathies, metabolic syndromes, mitochondrial disorders, and malignancies [2,3,4,5,6]. According to recent research, hyperactivation or inactivation of the phosphatase proteins can result in malignant tumors [7]. Efforts have been made in the hunt for better treatments, resulting in the identification of various kinase inhibitors that have been approved for clinical therapy [8]; however, success for more potent phosphatase inhibitors is still on the horizon of innovative therapeutics. The protooncogene PTPN11, which codes for Src homology 2-containing protein tyrosine phosphatase 2, or SHP2, is a crucial member of the protein tyrosine phosphatases (PTP) family [9,10].

**Figure 1:**
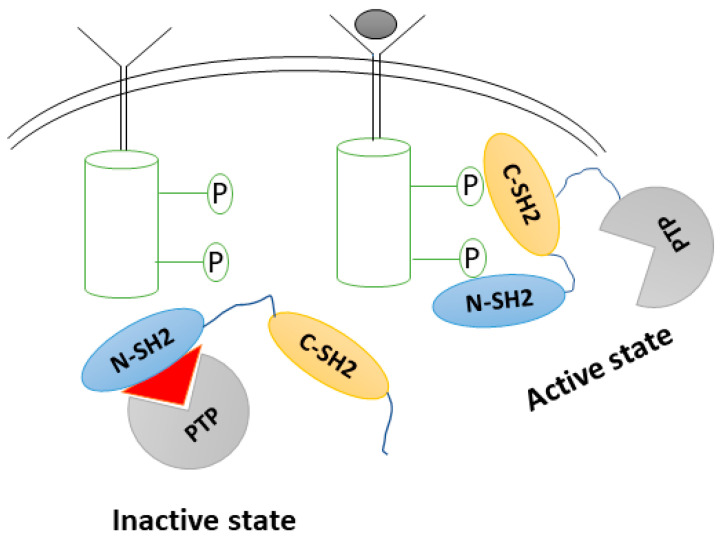
SHP2 activation mechanism in the presence of a substrate.

SHP2 is a 68 KDa protein with 593 amino acids residues that include two tandem SH2 domains (N-SH2 and C-SH2) of 1–120 residues; a catalytic protein tyrosine phosphatase domain (PTP, 221–225); and a disordered C-terminal tail (526–593 residues) containing Tyr542 and Tyr580 phosphorylation sites [11]. In its inactive state, SHP2 is autoinhibited by residues on the catalytic surface of the PTP domain and the N-SH2 domain, suppressing the protein’s activity and limiting substrate access to its catalytic site. When activated by growth factors, phosphopeptide binding causes an intramolecular “dissociation,” unblocking the N-SH2 domain from the PTP domain. In the event of a catalytic site blockage, the N-terminal SH2 immediately blocks its active site [11,12] (Figure 1).

SHP2 is well known for its extensive role in RAS-MAPK, PI3K-AKT, JAK-STAT, and associated signalling pathways [13,14]. Because of its involvement in signalling, it has been found to interact with other oncogenic drivers, particularly those of the RAS family, (KRAS) and so targeting SHP2 is critical for treatment of cancers associated with the different RAS oncogenes [5]. Gain-of-function mutations in SHP2 cancers, including childhood leukemia, have been demonstrated to affect mitosis, causing chromosomal instability and a heightened susceptibility to DNA damage [15]. From a glance at genetic syndromes, the *PTPN11* gene has been characterised as the most mutated in the RASopathy family of disorders, associated with several clinical conditions such as growth retardation cardiomyopathy, mental retardation, as well as predisposition to other forms of myeloproliferative disorders. Despite its regulatory role in cell cycle, proliferation, and differentiation, its involvement in dysregulation of mitochondrial homeostasis, endocrine, and energy metabolism is gradually gaining attention [16]. Tartaglia et al. found that leukemia caused by a gain-of-function SHP2-Y279C mutation decreases SHP2 catalytic activity [17]. The *PTPN11* gene SHP2-Y279C mutation causes alterations in cell function and has been associated with several clinical disorders. Gain of function SHP2-Y279C has been reported to produce hormonal/metabolic abnormalities via signalling pathway hyperactivation or inhibition. [18,19]. In animal studies, SHP2-Y279C hyperactivates the P13K/mTOR signalling, resulting in hypertrophic cardiomyopathy that can be reversed by rapamycin [19]. SHP2 is recruited in T cells and other immunosuppressive molecules such as CTLA-4, T-cell immunoglobulin, and ITIM domain protein (TIGIT) via unique phosphotyrosine patterns that regulate T-lymphocyte activation [20,21,22]. In light of this, blocking immunological checkpoints triggered by ligand–protein interactions has proven to be a potential strategy for identifying SHP2 targets in cancer immunotherapy [23].

SHP2 has become a fascinating focal point in understanding disease pathophysiology, and as such, it serves as a critical target in treating human disorders. Many attempts are being made to find high-efficacy inhibitors of SHP2 with minimal cell toxicity. This is the future of drug research and the treatment of human diseases. Traditionally, targeting the PTP domain would be required to inhibit SHP2 catalytic activity. In the past, a number of selective SHP2 inhibitors including PHPS1 and NSC-87877 have been studied in leukemia-associated SHP2 mutants [24,25]; however, many of these compounds still suffer from low bioavailability or permeability. Of course, targeting the PTP active site would necessitate highly selective molecules [26]; while efforts to find molecules with high specific binding to the PTP active site are in progress, alternative strategies involving targeting other sites (allosteric) of the SHP2 protein have been quite promising. Chen and colleagues revealed that SHP099 interacts with the C-NH2, N-SH2, and PTP domains via an allosteric manner [27], inhibiting RAS-ERK signaling and proliferation in leukemic mice. In addition, combining SHP244 and SHP099 has been demonstrated to improve cancer cell line inhibition by downregulating DUSP6 mRNA (a downstream MAPK pathway marker) [28].

Camptothecin (CPT), a pentacyclic alkaloid initially discovered from *Camtotheca acuminata* decades ago, has been in clinical trials since the late 1990s [29,30,31]. In the past, it was combined with corticosteroids to treat bladder cancer and certain leukemias. Its primary mode of action is to target the cell cycle by interfering with topoisomerase I activity. Because the natural CPT isomers’ main target is topoisomerase 1, while they were efficient in reducing tumor proliferation, their side effects were highly concerning due to considerable cellular toxicity. In light of this, novel CPT analogs have been developed in recent years, most of which are currently in clinical trials and have proven to be extremely successful in cancer therapy with minimal toxicity [32]. The goal is to target not only the topoisomerase enzyme, but also oncogenes involved in cancer proliferation and survival.

Based on previous findings that survivin is a crucial molecule at the intersection of cancer cell survival, division, and apoptotic regulation, recent efforts have resulted in developing novel camptothecin analogues such as FL118 through survivin gene expression in tumors [33,34,35]. Despite advances in the pursuit of specific potent inhibitors against Ptpn11-associated disorders, the biochemical interaction of derivatives of CPT (belonging to a family of quinoline hydrazine compounds) with the catalytic domain of Protein tyrosine phosphatase, SHP2 (Ptpn11) remains unexplored.

In that perspective, this study aims to provide in silico rationale for the recognition and binding of the camptothecin analogs; FL118 (also known as a survivin inhibitor) and irinotecan with the catalytic domain of Protein tyrosine Phosphatase-SHP2 (PTPc-SH2-SHP2, chain A). Hence, we performed molecular docking of FL118 and irinotecan with PTPc-SH2 wildtype (SHP2-WT) and in silico generated PTPc-SHP2 mutants (SHP2-Y279C and SHP2-R465G) and analyzed the ligand–protein interactions.

## 2. Results and Discussion

### 2.1. Physicochemical Characterisation of Ligand

**Figure 2:**
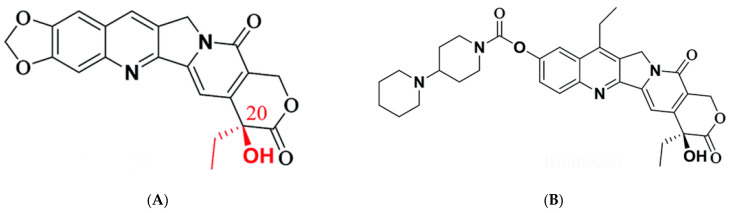
(**A**): 2D view of FL118; (**B**): 2D view of Irinotecan.

Physical characteristics of molecules determine not only bioavailability but also solubility and penetration of drugs across the various surfaces of human tissues [36,37]. Implementing the “rule of 5” [37], the camptothecin analog FL118 [10,11-Methylenedioxy-20(RS)-camptothecin] was predicted to have a LogP value < 5 (1.89), indicating a better solubility and hence a greater absorption and bioavailability compared to Irinotecan (4.10). Another vital feature predicted is the biological activity exerted by the compounds on their target, termed bioactivity [38,39]. It is feasible to classify and assume the best biological functional activity of a compound based on the bioactivity score assigned to it. FL118 is predicted to be moderately bioactive −0.20 (scores between −5.0 and 0.0) as an ion channel modulator and actively bioactive (scores > 0) as an enzyme inhibitor, GPCR ligand, or kinase inhibitor. The highest score prediction (0.99) depicts FL118 as an enzyme inhibitor (Table 1). Using Molinspiration server, the 2D structure, calculated physical properties, and predicted bioactivity of the camptothecin analog, [10,11-Methylenedioxy-20(RS)-camptothecin] (FL118) and irinotecan are presented below (Table 1; Figure 2A,B).

### 2.2. Prediction of Structure–Activity Relationship: ADMET of Compounds, FL118, and Irinotecan

Using SwissAdme, we predicted the pharmacological properties of the FL118 and irinotecan [40]. The BOILED-Egg model provides accurate prediction of pharmacokinetics and behaviour of the small molecules via computing the lipophilicity and polarity of molecules [41]. This model reveals both compounds are able to cross the skin and the human gastrointestinal track but not the blood–brain barrier. Albeit both compounds are permeable to the skin, FL118 shows slightly higher permeability (LogKp = −7.59) compared to irinotecan with logKp of −7.22. 

Generally, the solubility prediction of compounds is performed through different methods with the output of LogS. The SILICOS-IT method employs fragment base approach to calculate logs unlike the other methods which are based on a complete molecular topology [42,43]. FL118 falls in the moderately soluble class (LogS <−5.55) and irinotecan was observed to be poorly soluble (LogS = −7.28). Irinotecan violates the Ghose rule of druglikeness but obeys Lipinski’s rule, with, however, a single violation. Unlike irinotecan, FL118 obeys all the five rules of druglikeness [37,40]. Both compounds were found to have a bioavailability score of 0.55, implying that for every gram of compound ingested orally, 55% has been absorbed. The LogP value is a determining factor for a compound’s absorption, distribution, and penetration across membranes and barriers. According to Lipinski’s rule of 5, the LogP of a compound intended for oral administration should be <5. Both compounds were observed to be suitable for oral administration; however, FL118 is projected to be more lipophilic (LogP = 2.06) than irinotecan (LogP = 3.73) (Table 2A).

Toxicity which is given by LD50 is defined according to the globally harmonised system of classification with values assigned mg/kg. Employing the Pro TOX-II server for prediction of compound toxicity [44], FL118 belongs to Class III, toxic if swallowed (50 < LD50 ≤ 300), with a predicted LD50 of 50 mg/kg, while irinotecan is predicted class IV, harmful if swallowed (300 < LD50 ≤ 2000), and an LD50 of 765 mg/kg. Studies have shown that mutations in SHP2 can exhibit profound effects on mitochondria metabolism [16]; our attention is therefore brought to the involvement of these compounds in the mitochondrial and stress-related processes. Our model findings reveal that FL118 is a non-toxic target for mitochondrial membrane potential (MMP), whereas irinotecan is a toxic target for MMP (Table 2B). Both FL118 and irinotecan are able to inhibit certain complexes of the cytochrome system; FL118 inhibits CYP1A2, CYP2C9, CYP2D6, and CYP3A4, and irinotecan acts on CYP2C9, CYP2D6, and CYP3A4.

### 2.3. Mutagenesis In Silico

The wildtype crystal structure of human PTPc-SHP2 (PTPN11) with an accessible active site (PDB ID: 3B7O) was obtained from the protein database [45] and modelled using Molegro viewer, while the mutants were produced using Biovia Discovery studio programme. Residues 279 and 465 of PTP-conserved domain of SHP2 (chain A) were mutated from Tyrosine (aromatic) to Cysteine (neutral polar) and Arginine (basic polar) to Glycine (nonpolar), respectively. SHP2-R465G was generated as a novel mutant and the residues mutated in both SHP2-Y279C and SHP2-R465G, were among the predicted residues in the proteins’ binding pocket (active site) (Table 3). ARG465 was previously identified as a polar residue in the catalytic pocket of tyrosine phosphatase that acts as a ligand binding site and is responsible for SHP2 enzymatic activity [25]. Arg is well known for playing an essential role in biological processes such as growth and cell proliferation due to its immediate availability to be transformed into intermediates that replenish the Krebs cycle. Its involvement in metabolism and illnesses such as cancer has been studied. Iron chelators, according to Alicja et al., significantly inactivate and bind to the active sites of PTP1B and SHP2, interacting with arginine residues necessary for enzyme activation [46]. Furthermore, tyrosine residues have been shown to be vital in the regulation of biological pathways [47], as the phosphorylation of tyrosine residues often creates binding sites for other signalling proteins prompting interactions with SHP2 [48,49].

### 2.4. Active Site Recognition in Structures

**Figure 3:**
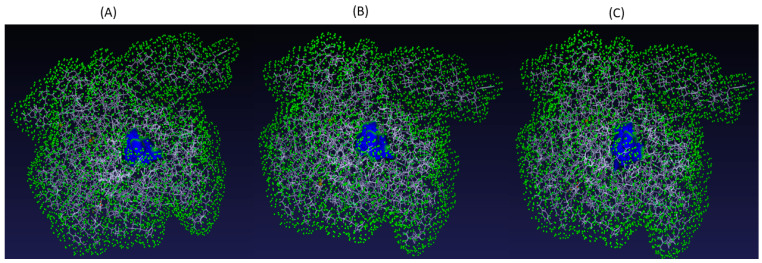
3D-visual of predicted binding pocket for (**A**) SHP2-WT, (**B**) SHP2-Y279C, and (**C**) SHP2-R465G (shown in blue color). The green dots only represent boundary planes which helps to better visualize the entire proteins’ structure in a 3D space.

Residues forming the proteins’ binding pockets (Table 3) were identified using PrankWeb server and modelled with PyMol software (Figure 3).

### 2.5. Physicochemical Characterisation of SHP2-WT, SHP2-Y279C and SHP2-R465G Mutant Structures

The physicochemical features of the SHP2-WT and mutant structures were computed by Expasy’s ProtParam server. The calculated pI value for SHP2-WT, SHP2-Y279C, and SHP2-R465G revealed a slightly acidic nature (pI < 7). The extinction coefficient was determined (45,630–45,630 M^−1^cm^−1^) based on the molar extinction coefficients of tyrosine, tryptophan, and cystine residues as a measure of how much light is absorbed by the protein at a specific wavelength. The aliphatic index, a positive indicator of globular protein thermostability, was high, indicating that both the wildtype and mutant proteins are stable over a wide temperature range. However, the proteins might be unstable (instability index > 40) due to the occurrence of certain dipeptides along the enzyme length that significantly differ. The negative values of GRAVY (Grand Average of Hydropathicity) infer that these proteins could likely interact better with water [50] (Table 4). The GOR4 server results revealed that random coils dominated the secondary protein structures, followed by alpha helix and extended strand, with no beta bridge or turn (Table 5).

### 2.6. Modelling and Assessment of Target Protein Structures

**Figure 4:**
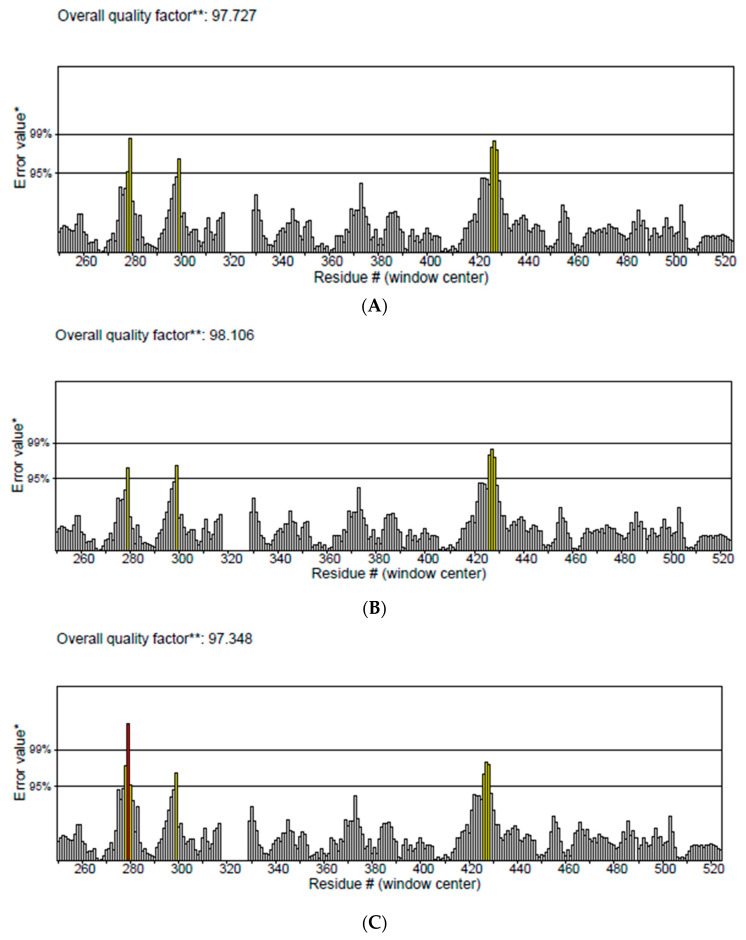

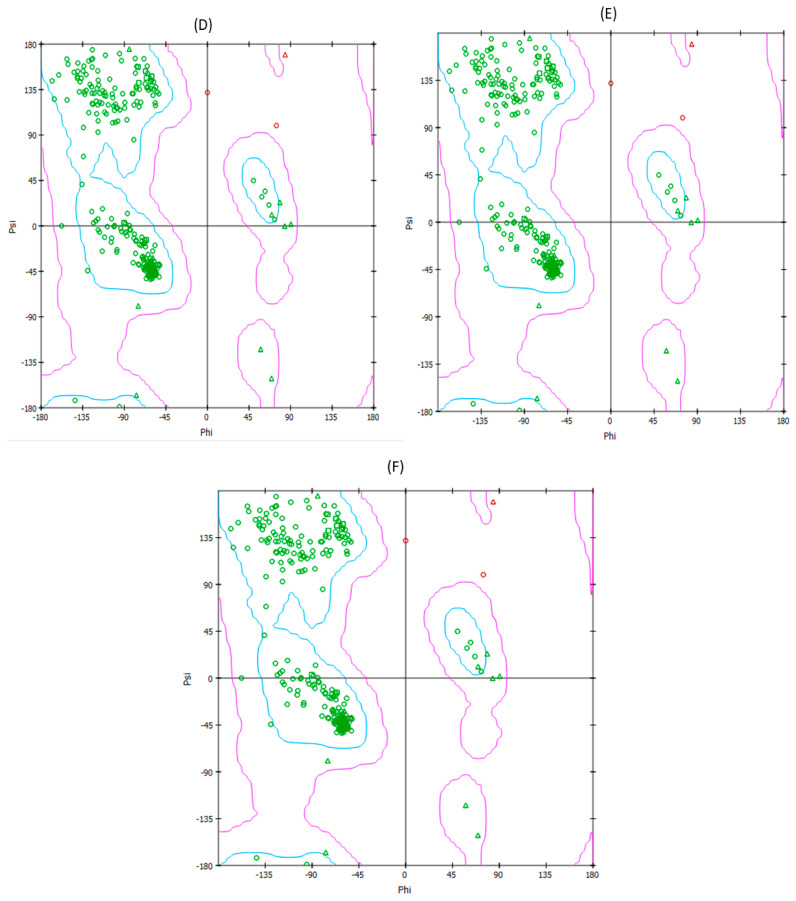
(**A**). ERRAT plots for (**A**) SHP2-WT, (**B**) SHP2-Y279C, and (**C**) SHP2-R465G structures. The misfolded region is indicated by yellow bars, located far from the active sites of the proteins. The grey bars represent the error zone between 95 and 99 percent, whereas the white regions reflect a lower error rate for protein folding. The two horizontal lines on the * error value axis reflect the confidence with which it is likely to reject regions that exceed the error value. ** expressed as the percentage of the protein for which calculated error value falls below 95% rejection limit. Good high-resolution structures generally produce values around 95% or higher. For lower resolutions (2.5–3 Å) the average overall quality factor is around 91%. (**B**): Ramachandran plots for (**D**) SHP2-WT, (**E**) SHP2-Y279C, and (**F**) SHP2-R465G with axis, Psi, and Phi angles (degrees). Each green dot represents an amino acid. Most residues cluster densely within the favorable regions (blue reference regions, upper and lower left quadrants) for acceptable proteins’ secondary structures.

The quality of the protein models, SHP2-WT, SHP2-Y279C, and SHP2-R465G was validated using the Verify 3D and ERRAT servers. ERRAT was used to examine non-bonded interactions between distinct atom types, with higher scores (>95 percent) indicating higher structure quality [51]. The ERRAT score for SHP2-Y279C was the highest (98.106%), followed by the wildtype and SHP2-R465G structures with close scores of 97.727% and 97.348%, respectively (Figure 4A–C). Albeit none of the residues exceeded the 99 percent threshold error value, they are all within the generally acknowledged range (>95 percent) of suitable high-quality model structures [51,52]. This data was further supported by the Ramachandran plots, which showed that the majority of amino acid residues were within the favorable region of protein secondary structures (Figure 4D–F). Thus, this analysis reveals that all conformations of modelled structures fit in spectrum of high-quality models.

### 2.7. Molecular Docking of FL118 and Irinotecan to PTPc-SHP2-Wildtype (SHP2-WT); and FL118 to Mutant SHP2 Structures

The camptothecin analog FL118 binds to the active site of PTPc domain of SHP2-WT with a lower negative scoring value of −7.54 Kcal/mol compared to irinotecan (−6.85 Kcal/mol), indicating that FL118 has a stronger binding affinity for our protein of interest. The affinity energies, however, did not correlate with the intermolecular energies since irinotecan-SHP2-WT formed a slightly lower negative intermolecular energy (−8.64 Kcal/mol) than FL118-wildtype motif (−8.13 Kcal/mol). Other factors such as van der Waals forces, dissolvation energy, and electrostatic energy may have contributed to the slight decreased negative intermolecular energy of the irinotecan–protein motif. Because the FL118-SHP2 wildtype motif had a higher binding affinity, we next docked FL118 with SHP2 mutants to fully understand the binding strength and extent of binding of FL118 to SHP2 protein harboring mutations, as well as the influence these mutants may have on the binding affinity. Intriguingly, FL118 binds to the SHP2-Y279C mutant with a lower negative scoring value (−6.94 Kcal/mol) than irinotecan-SHP2-WT (−6.85 Kcal/mol), showing that FL118 has a higher binding affinity for the protein despite the presence of a mutation (Table 6). The slightly larger negative energy values for SHP2-Y279C and SHP2-R465G motifs relative to FL118-wildtype motif are associated with the change in active site structural conformation caused by the mutation. Though the difference in affinity energies between SHP2-Y279C and SHP2-R465G is insignificant, the effect of SHP2-R465G mutation creates a weaker binding interaction pocket for the ligand. It is noteworthy that the estimated affinity energies were calculated from the sum of the intermolecular and torsional energy. Another important binding affinity determinant of the ligand–target interaction is the inhibition constant, Ki, defined by its inverse relationship with binding affinity [53,54]. Depending on the type of inhibition (competitive, non-competitive, or uncompetitive), this value is numerically equal to IC_50_ or one-half of IC_50_ [55]. Our findings show that the FL118–mutants and irinotecan–protein complexes had higher KI values (8.14, 13.12, and 9.53 µM, respectively) than the FL118-SHP2-WT complex (3.00 µM) (Table 6). This suggests that a lower dose concentration of FL118 will be required to inhibit the wildtype protein, as opposed to mutant proteins, which will require a greater dose of FL118. Similarly, as compared to the FL118 dose required to produce an effect on the wildtype protein, a substantially greater dose of irinotecan will be required to produce the same effect on the wildtype protein. Taken together, the lower negative binding energies of FL118-SHP2-WT and FL118-Y279C mutant structures indicate that FL118 analog has a stronger binding affinity and inhibitory effect on the protein’s active site PTPc domain. In addition, it suggests that the protein was in a favorable state during contact with the ligand.

**Figure 5:**
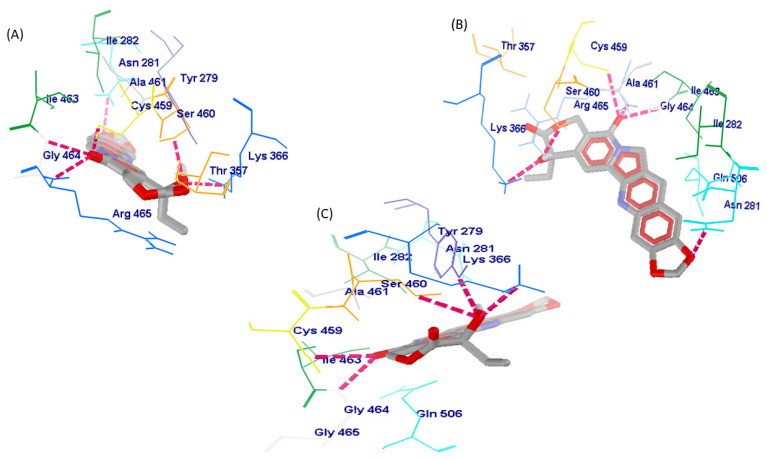
Docking view of (**A**) SHP2-WT, (**B**) SHP2-Y279C, and (**C**) SHP2-R465G mutants with FL118 ligand. Hydrogen bond interactions are represented in pink lines; depending on the amino acid type, the interacting residues are shown in different wireframe colors. The grey-red structure in thick-stick graphical style represents the Ligand.

**Figure 6:**
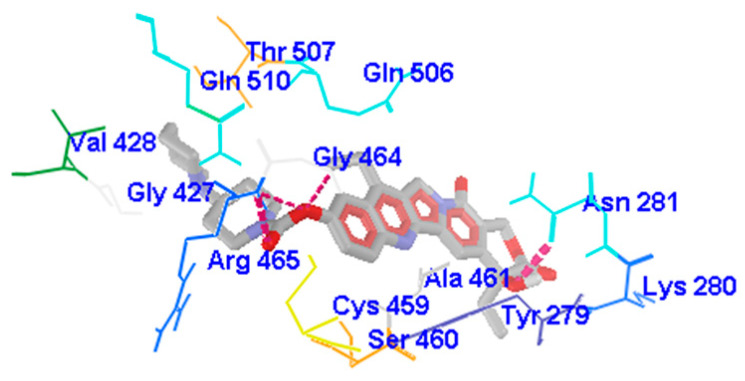
Docking view of SHP2-WT with Irinotecan. Hydrogen bond interactions are represented in pink lines; depending on the amino acid type, the interacting residues are shown in different wireframe colors. The grey-red structure in thick-stick graphical style represents the Ligand.

Hydrogen bonds formed between interacting entities contributes to the stability of the complex generated; the greater the number of hydrogen bonds, the more stable the complex [56,57]. The results show that SHP2-WT, SHP2-Y279C, and SHP2-R465G were stabilized by six, five, and four hydrogen bonds, respectively; however, irinotecan-SHP2-WT was stabilised by only two hydrogen bonds (Table 7 and Figure 5 and Figure 6). The residues that formed H-bonds between ligand–protein complex includes the following: ASN281, LYS366, SER460, GLY464, ARG465, CYS459, for SHP2-WT complex; LYS366, SER460, GLY464, ARG465, CYS459, for SHP2-Y279C complex; TYR279, LYS366, SER460, and GLY464 for SHP2-R465G complex and ASN281 and ARG465 for irinotecan–protein complex. This data points to a much weaker interaction between irinotecan and wildtype SHP2 than the FL118–SHP2 interaction complex (including mutants), suggesting a less stable irinotecan–SHP2 complex. In both mutants and wildtype active sites, LYS366, SER460, and GLY464 were common amino acid residues targeted by the FL118 ligand. All residues except ASN281 (side chain residue) that participated in H-bond formation were among residues forming the binding pocket of the SHP2 protein, suggesting the inhibitor’s specificity for the active site PTPc domain and the formation of very stable complexes (Figure 5 and Figure 6). This was particularly observed in SHP2-R465G, in which all the residues involved in H-bond formation were among the predicted active site residues. The binding of irinotecan to the active site of SHP2 protein is stabilized by two hydrogen bonds formed (by residues ASN281 and ARG465), with only ARG465 belonging to residues forming the protein’s binding pocket, whilst ASN281 does not. Hence, this leaves the irinotecan–SHP2 complex with a single hydrogen bond in the protein’s active site. The majority of active site interacting residues (CYS459, GLY464, ALA461, TYR279) in the irinotecan–SHP2 complex, on the other hand, contributed to the development of weaker intermolecular forces, primarily Pi hydrophobic interactions. Taken together, our findings suggest that FL118 ligand forms a more stable and selective binding to SHP2 active site than irinotecan.

The shortest bond distances in the protein’s binding pocket were formed between CYS459 and FL118 atom O29 (1.78 Å), H35 of FL118 and SER460 (1.86 Å), and GLY464 and FL118 atom O29 (1.91 Å) (Table 7). This infers to strongest and most stable bond formed between these residues and the corresponding ligand atoms in comparison to other hydrogen bonds with greater bond lengths. Noteworthily, most H-bonds were formed between O29 acceptor atom (of the quinoline rings) and corresponding amino acid residues (particularly in SHP2-WT and SHP2-Y279C), suggesting the ring structure to which atom O29 belongs is critical for the dynamics of FL118–SHP2 interactions. Thus, suggesting a modification of this ring structure might change (increase or decrease) the binding energies as well as the binding affinity of the ligand–protein complexes.

Considering the efficiency of binding and selectivity of FL118, preclinical studies of novel FL118 have proven quite successful as survivin inhibitors in patients with multiple myeloma, given its high efficacy in relapsed/refractory patients and patients with P53 dysfunction [58]. Not only does it interfere with survivin, but it also interferes with other apoptotic genes including Mcl-1, XIAP, and cIAP2 [59]. FL118 inhibits survivin with a greater efficiency than those with DNA topoisomerase I [60]. A study by Wang and colleagues demonstrated that cancer stem cells (CSCs) are more sensitive to FL118 than cisplatin by downregulating CSC markers such as ABCG2, ALDH1A1, Oct4, and drug-resistant proteins P-gp and ERCC1. In addition, FL118 reduced CSC invasiveness [61], hence making FL118 an ideal candidate against drug resistance and cancer metastasis. A recent study on nude mice harboring the ES-2 ovarian cancer cell line demonstrates that FL118 has a greater anti-tumor impact than topotecan (a camptothecin analog). Furthermore, FL118 inhibited cell proliferation by upregulating the Cytoglobin (CYGB) protein [62].

### 2.8. Model Simulation and Evaluation of FL118-SHP2 Wildtype Complex

**Figure 7:**
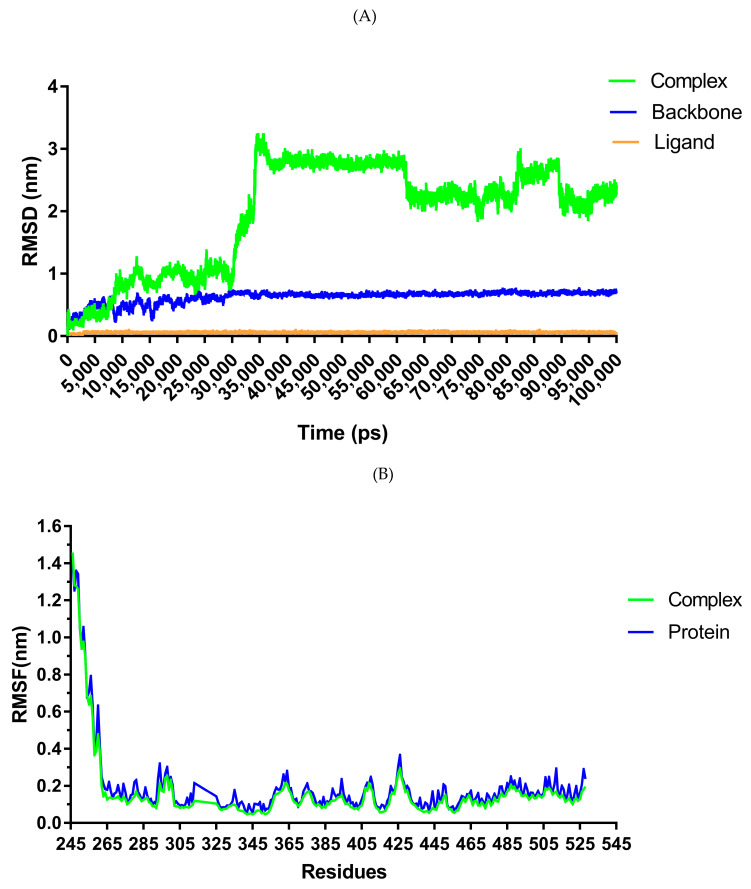

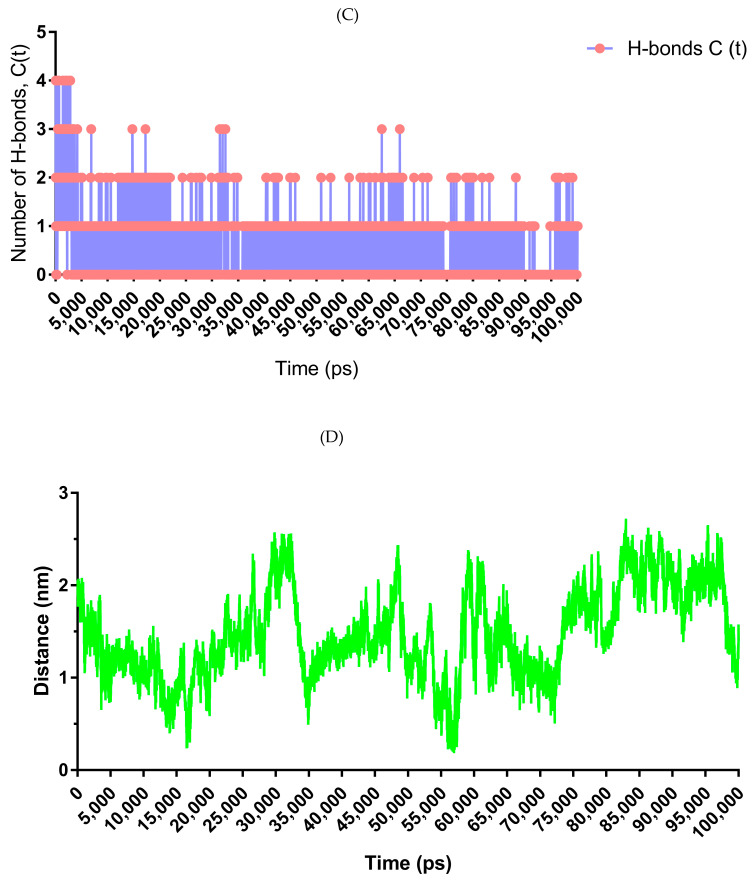
The dynamics of FL118-SHP2 complex motif: (**A**) RMSD of the ligand–protein complex (green), protein backbone (blue), and ligand (orange); (**B**) RMSF of the C-alpha amino acid for each residue in the complex (green) and the protein before complex formation (blue); (**C**) Hydrogen bond correlation over the simulation time period; (**D**) Salt bridge formation FL118-SHP2 wildtype motif over time.

Given the high affinity of FL118 to SHP2 protein, we further performed a 100 ns (100,000 ps) molecular dyanamic (MD) simulation run for the FL118-SHP2 wildtype complex motif. Figure 7A shows the root mean square deviation (RMSD) value of the protein-ligand complex over the simulation time. The RMSD provides insights into the structural conformations throughout the simulation. A deviation was detected in the complex (30,000–35,000 ps) during the initial phase of simulation up to 35,000 ps (RMSD = 3.17 nm). Beyond 35,000 ps, the system starts to converge, achieving stability as it approaches 100,000 ps (with high stability from 60 to 100 ns). The sharp divergence (between 30,000 and 35,000 ps) may imply that the protein underwent some structural change during that time period, although it is unclear if the ligand is the sole reason for this variation.

The root mean square fluctuations (RMSF) of the alpha carbon amino acid for each contact residue were calculated using the FL118-SHP2 trajectory data displayed in Figure 7B. In this analysis, all residues in the protein model fluctuated between 0.046 and 0.295 nm throughout the entire simulation. Among the residues in the protein’s active pocket, residue 425 (ASP) had the greatest fluctuation (0.2683 nm), followed by residue 427. (GLY). RMSF of the protein before complex formation did not differ considerably from that after complex formation. The only significant change was observed at residues 313, 325, and 326 of the protein before complex formation. Other slight changes could be observed at residues 271–274, 335,447, 508, 512, and 523. Surprisingly, none of these residues belong to the residues that form the protein’s binding pocket.

The autocorrelation function assessed the probability of a hydrogen bond being present (broken or reformed within a given time interval) at time (t), assuming it was present at time zero. The overall lifetime analysis of hydrogen bond formation between the protein and the ligand FL118 indicated that the complex was stabilized by one to two hydrogen bonds by a significant number of protein residues throughout the simulation period. Three H-bonds stabilized the structure from the start of the simulation (0 ps) until 65,970 ps, and four H-bonds only for 2810 ps. The creation of up to 6 H-bonds by some residues (as seen in Table 7, during docking) is likely to have been broken within a few ps of the simulation start time. However, it is uncertain whether this is why the simulation failed to detect the signals for the development of six H-bonds (Figure 7C).

Salt bridges are also required for protein structural stability. The smaller the distance between residues involved in the production of salt bridges, the greater the complex’s stability. The simulation calculations revealed that the FL118-SHP2 complex achieved stability at a minimum periodic distance ranging from 0.198 to 2.662 nm throughout the experiment, with the shortest distance (0.198 nm) recorded at time 56,930 ps (Figure 7D). Taken together, the binding interaction between FL118 and SHP2 proteins reaches stability after 34,000 ps. ASP425 and GLY427, two residues in the protein’s binding pocket, do not stably interact with the ligand over time as seen by their significant fluctuation levels. Furthermore, during this simulation period, our ligand–protein complex is mostly stabilized by two or one H-bond.

Furthermore, following the simulation of FL118-SHP2-WT, we employed MMPBSA [63,64] calculations to determine the average binding energy for the complex during the stabilization phase (60-100ns). Within the timeframe from 60-100ns, we successfully generated a total delta binding energy of -13.24 kcal/mol for 60-70ns time window. However, after 70ns (up to 100ns), MMPBSA was unable to generate the total average energy due to “some undefined energy terms”. Though the reason for this remains unclear, we speculate an unfavorable conformational change in protein structure (after 70ns of the simulation run time) might have resulted in the unsuccessful generation of the total average binding energy.

Lastly, we checked if the ligand takes a stable position in the active pocket after 100 ns of simulation. We found that two hydrogens bonds (formed between the ligand and GLN257) maintained the stability of the complex after 100 ns of simulation unlike before simulation (after docking analysis), which was stabilized by six hydrogen interactions. Residues involved in the interaction between the ligand and protein after 100 ns include LEU261, GLN257, LEU262, GLU258, LEU254, GLN255, PHE251, and TRP248. It is noteworthy that the residue (GLN257) which formed the hydrogen interactions was not found to be among residues forming the predicted binding pocket of the protein, indicating a shift of the ligand’s position in the binding pocket of the protein after 100 ns of simulation.

**Figure 8:**
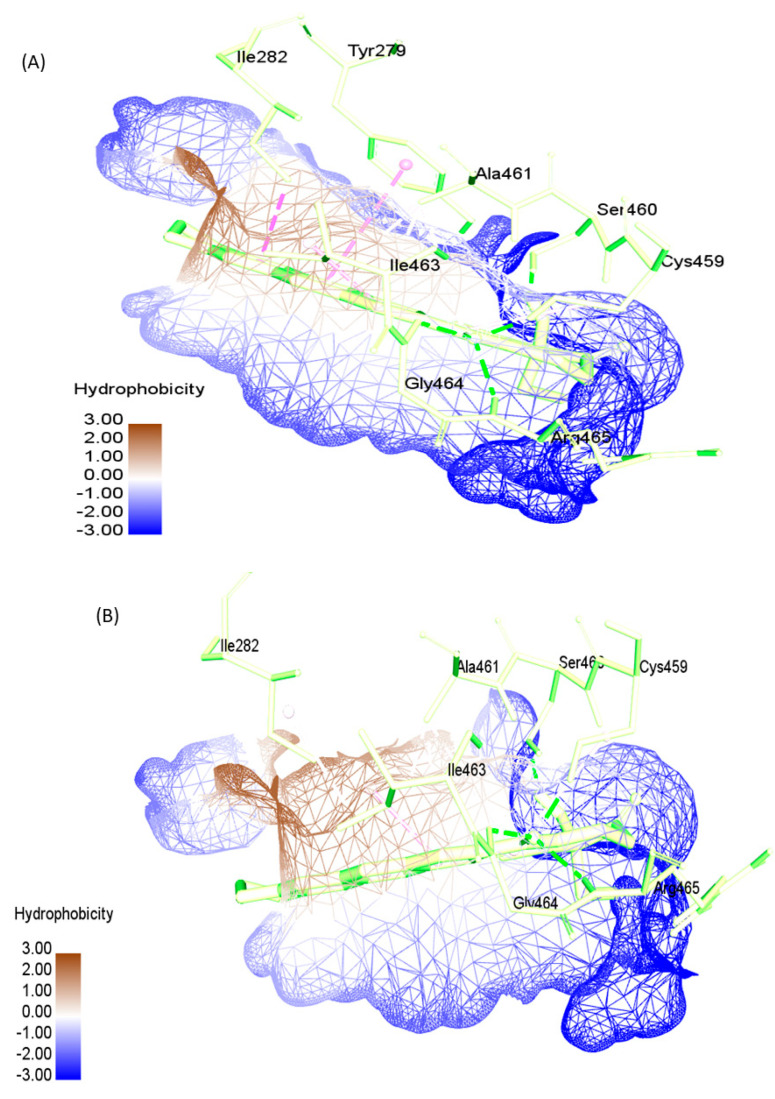

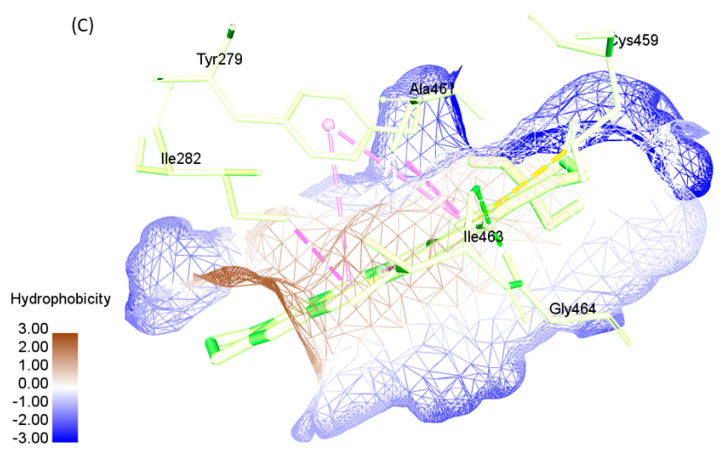
Projection of complexes’ hydrophobicity. (**A**) PTPc-WT, (**B**) SHP2-Y279C, and (**C**) SHP2-R465G mutants and FL118 complexes, with hydrophobic surfaces represented in brown and hydrophilic surfaces in blue.

Another fundamental type of interaction in protein–ligand complexes involves hydrophobic interactions. Hydrophobic and polar interactions do not just steer up the thermodynamic stability of complexes but also the mechanical stability [65,66]. Ferenczy and Kellermayer observed that hydrophobic interactions contribute about one-fifth to one-third of the total force field in a protein [65]. Our findings also revealed the hydrophobic and hydrophilic surfaces, as well as the interacting residues (Figure 8). In SHP2-WT and SHP2-R465G, three types of hydrophobic contacts were found including Pi hydrophobic, Alkyl hydrophobic, and mixed Pi/Alkyl hydrophobicity. In SHP2-Y279C, we observed just the Alkyl and mixed Pi/Alkyl hydrophobicity. In all three complexes, residues ILE282 and ILE463 are the primary residues forming most of the hydrophobic contacts (Figure 8).

## 3. Materials and Methods

### 3.1. Ligand Structure and Characterization

The Simplified Molecular Input Line Entry System (SMILE) for compounds FL118, [IUPAC (4R)-4-Ethyl-4-hydroxy-8,9-methylenedioxy-1H-pyrano [3′,4′:6,7] indolizino [1,2-b] quinoline-3,14(4H,12H)-dione], and Irinotecan were obtained from public database [67] and the 2D/3D structure generated using Molinspiration Cheminformatics [37] and CORINA CLASSIC 3D servers required for further docking processing. In addition, their physiochemical properties and predicted bioactivity were calculated using these tools.

### 3.2. ADMET Profiling of Both Compounds Was Performed by Employing SwissAdme and Pro Tox-II Webservers

SwissAdme/Pro Tox-II servers applies serveral in silico methods and models to predict the behaviour of compounds (ligands) and their potential as drug candidates. These servers applied Lipinski’s rule of five and other computational modelling techniques such as quantitative structure-activity relationship (QSAR) to calculate the molecualar properties of the compounds including lipophilicity (LogP), water solubility, polarity and molecular weight which is used to predict the ADME and toxicity of compounds on targets. Also, these tools employed machine learning and AI algorithms that trains large datasets with known information to generate predictive models.

### 3.3. Target Structures

The tertiary structure of human SHP2 (PTPN11) was obtained from public database. The complete sequence for the PTPc domain contained 272 amino acids as retrieved from RCSB Protein Databank (PDB: 3B7O). The 3D structure was viewed with Molegro Molecular Viewer [68].

### 3.4. In Silico Mutagenesis

Mutations of 3B7O (PTPc-SH2-SHP2) were created computationally at residues 279 and 465 using the BIOVIA Discovery studio software [69]. These residues play a vital role in the enzymatic activity of the SHP2 protein and location in the active site of the protein’s conserved catalytic domain.

### 3.5. Active Site RECOGNITION

Binding sites of protein structures were identified using PrankWeb server [70].

### 3.6. Model Simulation and Assessment

The stereochemical quality and accuracy of structures were validated with Verify 3D and ERRAT server [51]. Using the Discovery studio software, the Ramachandran plots was generated to confirm that most residues lie within the generally accepted region for secondary protein structures [69].

### 3.7. Molecular Docking

To comprehend the molecular interaction between camptothecin and SHP2-WT and mutants, a flexible small molecule rigid protein docking was performed with AutoDock Version 4.2 [71]. In preparing the protein structures for docking, water molecules and any available heteroatoms were removed, only polar hydrogens added, and the Kollman charges added to structures. Likewise, in imputing the ligand molecule, hydrogens and charges were automatically added to prepare the ligand. Torsion bonds were also checked for each ligand type and kept within the required limit (max 32). The Grid box axis parameters were set to the active site of protein, enabling specific ligand target to active site. This grid X,Y,Z-center coordinates were generated using data predicting proteins’ binding pocket from the binding site prediction server (PrankWeb). Furthermore, the docking parameters including the genetic algorithm were set to n = 50 runs and a population size of 300 for all ligand–protein complexes. The same docking simulation performed for SHP2-WT was also applied to the single point mutants (SHP2-Y279C and SHP2-R465G).

### 3.8. Docking Analysis of Ligand-Protein Complex

Further analysis and view of ligand-protein complex structures was carried out using a combination of servers and software including Proteins Plus [72], which allowed visualization of 3D complex structures and creation of 2D pose depictions; the LIGPLOT programme [73] for automatic generation of 2D ligand-protein interaction diagrams. It is run from an intuitive java interface that allows on-screen editing of the plots; Molegro Molecular viewer [68] visualises the 3D ligand–protein complex, generating the interaction surfaces. The BIOVIA Discovery studio [69] creates a surface next to the ligand to analyse the hydrophobicity in a protein–ligand complex. These protein (receptor) surfaces offer a unique window into how a receptor functions. The hydrophobicity density map, which was created using the Kyte-Doolittle scale and depicts the hydrophobic and hydrophilic regions in brown and blue, is one of the receptor atoms attributes that gives the surface its color.

### 3.9. Molecular Dynamics Simulation for LIGAND–Protein Complex

The docked FL118-SHP2 wildtype complex was used to perform the MD simulation. The GROMACS package 2023.1 adopting the CHARM27 all-atoms forcefield parameter was carried out to run MD simulation [74]. The protein structures were solvated in triclinic box, using periodic boundary conditions and the SPC (simple point charge) water model. The ligand topology file was generated with the help of SwissParam server [75] and Chimera software [76] to include heteroatom due to limitations of GROMACS to parameterize the heteroatom group in PDB file. Conjugate gradient algorithm was executed for every 1000 steps of steepest descent minimization. The system was equilibrated at a constant temperature (300 K) and pressure (1.0 bar) for 100 ps. Lastly, the equilibrated structures were subjected to molecular dynamic simulations for 100 ns (100,000 ps) with a LINCS algorithm 2 fs time step. The trajectory created from every pico second of the structural analysis was utilized to calculate RMSD, RMSF, H-bonds, and salt bridges formed between the protein and ligand in the docked complex.

## 4. Conclusions

In this study, we developed 3D mutant models (SHP2-Y279C and SHP2-R465) from PTPc-SH2-SHP2 wildtype protein obtained from Protein Data Bank. The structures were tested for quality, and the general geometry of the backbone conformation was found to be reliable to proceed with the study. This protein–ligand interaction was investigated to evaluate the recognition and binding of the novel camptothecin analogs, FL118, and irinotecan to the human PTP-catalytic domain of SHP2 protein. This is essential for leveraging potentially synergistic compounds (inhibitors), identifying critical interacting targets in *PTPN11*-associated illnesses, and improving anti-tumoral activity and reducing cell toxicity. The docking interaction between the analogs (ligand) and protein was calculated and analyzed with the Autodock 4.2 programme.

Several factors were considered in the docking including protonation states for residues, flexible side-chain minimization, and the inclusion of solvation effects following specific procedures while using the program. However, to achieve greater accuracy, more advanced scoring functions or more exhaustive sampling of the possible binding modes and flexibility can be used, though these modifications usually increase the computational cost. Additionally, in using Autodock program, genetic algorithms involve lots of time for a single energy calculation. The binding process usually involves a balance of many different physical interactions: a flexible ligand may gain favorable interactions while suffering a significant entropic penalty as a result of binding. Notably, no ideal software exists to date that accurately predicts how water molecules interact with target molecules.

Despite the constraints, computational approaches have been widely used in research involving ligand–protein and protein–protein interactions [77], yielding reliable results and experimentally proven results. As a result, we have summarized the most important findings from our analysis.

The FL118 analog was found to have lower negative affinity energies with PTPc-SHP2 wildtype (−7.54 Kcal/mol) and SHP2-Y279C mutant (−6.94 Kcal/mol) than irinotecan analog, which had a higher negative binding energy (−6.85 Kcal/mol), indicating a better (greater) binding affinity of FL118 analog to SHP2 protein. Furthermore, the interaction of FL118 with SHP2-WT, SHP2-Y279C, and SHP2-R465G structures was stabilized by six, five, and four hydrogen bonds, respectively, in contrast to the irinotecan-SHP2 wildtype complex, which was stabilized by two hydrogen bonds. LYS366, SER460, and GLY464 were common amino acid residues targeted by the FL118 ligand in both mutants and wildtype active sites with the maximum number of hydrogen bonds formed in the FL118-SHP2 wildtype structure, indicating a greater stability of complexes generated by FL118 ligand. The docking interactions of both ligands (FL118 and irinotecan) targeted residues in the predicted proteins’ binding pocket, suggesting a selective and specific binding of the analogs to protein’s active site.

Furthermore, RMSD, RMSF, hydrogen bonds, and salt bridge analyses were used to validate the protein–ligand complex structures. The findings proved that FL118 formed strong bonds and a stable complex with SHP2 protein. Reports from existing studies [58,59,61,62] strengthens the significance of FL118 over other camptothecin analogs in fighting cancer and related disorders. Together, our findings, which are based on computational analysis of the binding and interaction of FL118 with SHP2, paves the way for further in vivo and in vitro investigations including experimental validations such as site-directed mutagenesis or functional assays to confirm the binding site of the ligand. Therefore, not only should biochemical and in vivo studies of ligand interactions involving SHP2 (PTPN11) be carried out in the future, but also laboratory experiments that will be required to develop potential therapies against PTPN11 diseases as well as related disorders.

## Figures and Tables

**Table 1:** Compounds (ligands) physiochemical characterization and bioactivity prediction.

Ligands	Physical Properties						Predicted Bioactivity	
	miLogP	MW/gmol^−1^	Natoms	nON/nOHNH	Nrotb	Vol Cubic/Å	Bioactivity	Scores
FL118 [10,11-Methylenedioxy-20(RS)-camptothecin]	1.89	392.36	29	8/1	1	321.34	Ion channel modulator	−0.20
							Enzyme inhibitor	0.99
							GPCR ligand	0.41
							Kinase inhibitor	0.26
Irinotecan	4.10	586.68	43	10/1	5	530.67	Ion channel modulator	−0.45
							Enzyme inhibitor	0.54
							GPCR ligand	0.33

**Table 2:** (**A**). ADME of target compounds. (**B**). Toxicity prediction model for FL118 and irinotecan.

**(A)**						
	**Water Solubility** **LogS (SILICOS-IT)**	**Pharmacokinetics**			**Druglikeness** **(Lipinski’s)**	**Lipophilicity** **(Log Po/w)**
		**GI Absorption**	**BBB**	**LogKp** **(Skin Permeation) cm/s**		
**FL118**	**−5.5 (moderatly soluble)**	**High**	No	−7.59	Yes, 0 violation	2.06
**Irinotecan**	−7.28 (Poorly soluble)	High	No	−7.22	Yes, 1 violation: MW > 500	3.73
**(B)**						
**Classification**		**Target**	**Prediction**		**Probability**	
			**FL118**	**Irinotecan**	**FL118**	**Irinotecan**
**Organ toxicity**		Hepatotoxicity	Inactive	Inactive	0.82	0.67
**Stress response pathways**		Nuclear factor (erythroid-derived 2)-like 2/antioxidant responsive element (nrf2/ARE)	Inactive	Inactive	0.70	0.94
		Heat shock factor response element (HSE)	Inactive	Inactive	0.70	0.94
		Mitochondrial Membrane Potential (MMP)	Inactive	Active	0.65	0.76
		Phosphoprotein (Tumor Supressor) p53	Active	Active	0.51	0.60
		ATPase family AAA domain-containing protein 5 (ATAD5)	Inactive	Inactive	0.98	0.95
**Toxicity end points**		Carcinogenicity	Inactive	Inactive	0.54	0.61
		Immunotoxicity	Active	Active	0.99	0.99
		Mutagenicity	Inactive	Inactive	0.54	0.67
		Cytotoxicity	Active	Active	0.98	0.79

Active = toxic effect; Inactive = no toxic effect.

**Table 3:** Predicted binding pocket residues for SHP2-WT, SHP2-R465G, and SHP2-Y279C structures.

Protein	Probability Score	Residues Forming the Binding Pocket
**SHP2-WT**	0.776	TYR279, ILE282, THR357, GLU361, ARG362, LYS366, TRP423, PRO424, ASP425, GLY427, VAL428, CYS459, SER460, ALA461, ILE463, GLY464, ARG465,GLN506, THR507, ALA509, GLN510
**SHP2-R465G**	0.893	TYR279, ILE282, THR356, THR357, GLU361, ARG362, LYS366, TRP423, PRO424, ASP425, GLY427, VAL428, CYS459, SER460, ALA461, ILE463, GLY464, GLY465, PHE469, GLN506, THR507, ALA509, GLN510
**SHP2-Y279C**	0.782	ARG278, CYS279, ILE282, THR357, GLU361, ARG362, LYS364, LYS366, TRP423, PRO424, ASP425, GLY427, VAL428, CYS459, SER460, ALA461, ILE463, GLY464, ARG465, GLN506, THR507, ALA509, GLN510

**Table 4:** Predicted physicochemical behavior of SHP2 wildtype and mutants.

Protein	Length	Mol.Weight (Daltons)	pI	−R	+R	Extinction Coefficient	Instability Index	Aliphatic Index	GRAVY
**SHP2-WT**	272	31,748.06	6.55	38	36	45,630	46.95	76.91	−0.609
**SHP2-Y279C**	272	31,688.02	6.55	38	36	44,265	47.23	76.91	−0.595
**SHP2-R465G**	272	31,648.92	6.34	38	35	45,630	47.09	76.91	−0.594

**Table 5:** Secondary structures of SHP2-WT, SHP2-Y279C, and SHP2-R465G.

Secondary Structure	Random Coil (Cc/%)	Alpha Helix (Hh/%)	Extended Strand (Ee/%)	Beta Turn(Tt/%)
**SHP2-WT**	43.75	33.09	23.16	0
**SHP2-Y279C**	44.85	32.72	22.43	0
**SHP2-R465G**	43.38	28.31	28.31	0

**Table 6:** Docking results of ligands with wildtype SHP2-WT and mutants.

Ligands	FL118			Irinotecan
	SHP2-WT	SHP2-Y279C	SHP2-R465G	SHP2-WT
Affinity energy (binding affinity) (kcal/mol)	−7.54	−6.94	−6.66	−6.85
Ligand efficiency	−0.26	−0.24	−0.23	−0.16
Inhibition constant, Ki/µM	3.00	8.14	13.12	9.53
Intermolecular energy (kcal/mol)	−8.13	−7.54	−7.26	−8.64
Internal energy (kcal/mol)	−0.18	−0.17	−0.15	−1.59
Torsion energy (kcal/mol)	0.60	0.60	0.60	1.79
Unbounded Extended energy (kcal/mol)	−0.18	−0.17	−0.15	−1.59
Reference RMS (Å)	67.11	67.0	66.57	65.24

**Table 7:** Hydrogen bond interactions with bond distances between SHP2-WT, SHP2-Y279C, SHP2-R465G and FL118; SHP2-WT and Irinotecan.

Ligand	Protein	Donor Atom	Acceptor Atom	Distance (Å)
FL118	SHP2-WT	ASN281:HD	FL118:O20	2.93
		LYS366:HZ	FL118:O4	2.80
		FL118:H35	SER460:OG	1.90
		GLY464:HN	FL118:O29	2.14
		ARG465:HN	FL118:O29	2.20
		CYS459:HG	FL118:O29	1.78
	SHP2-Y279C	LYS366:HZ	FL118:O4	2.81
		FL118:H35	SER460:OG	1.86
		GLY464:HN	FL118:O29	2.08
		ARG465:HN	FL118:O29	2.17
		CYS459:HG	FL118:O29	1.92
	SHP2-R465G	FL118:H35	TYR279:OH	3.11
		LYS366	FL118:O4	2.74
		SER460	FL118:O4	2.79
		GLY464	FL118:O29	1.91
Irinotecan	SHP2-WT	Irinotecan:H54	ASN281:OD1	1.86
		ARG465:HN	Irinotecan:O29	2.05

## Data Availability

Not applicable.

## Abbreviations

BCR-ABL	Breakpoint cluster region-Abelson gene
CPT	Camptothecin
CSF1	Colony stimulating factor 1
CTLA	Cytotoxic T-lymphocyte-associated protein 4
DUSP6	Dual specificity protein phosphatase 6
GAB	GRB2-associated binding protein
GPCR	G-protein-coupled receptor
GRAVY	Grand Average of Hydropathicity
GRB2	Growth factor receptor-bound protein 2
H-bonds	Hydrogen bond interactions
ITIM	Immunoreceptor tyrosine-based inhibitory motif
JAK	Janus kinase
KI	Inhibitor constant
KRAS	Kirsten rat sarcoma viral oncogene homolog
MAPK/ERK	Mitogen-activated protein kinase/Extracellular signal-regulated kinase 1/2
MD	Molecular dynamics
mTOR	mammalian target of rapamycin
MW	Molecular weight
MW	Molecular weight
nOHNH	number of OH-NH bonds
nON	number of O-N bonds
nrotb	Number of rotational bonds
P13K/AKT	Phosphatidylinositol-3-kinase (PI3K)/Ak strain transforming
PDB	Protein data bank
pI	Isolectric point
PTK	Protein tyrosine kinase
PTP	Protein tyrosine phosphatase
PTPc-N11/PTPN11	Protein tyrosine phosphatase catalytic-Non receptor type 11
RMSD	Root mean spuare deviation
RMSF	Root mean spuare fluctuation
RS	Right/left
SHP2	Src tyrosine phosphatase protein
STAT3	Signal transducer and activator of transcription 3
TIGIT	T-cell immunoreceptor with immunoglobulin and ITIM domains
WT	Wildtype
